# Intention to Pay for Vaccination and Influencing Factors of General Residents: A National Cross-Sectional Study

**DOI:** 10.3390/ijerph191811154

**Published:** 2022-09-06

**Authors:** Weixin Zhang, Xin Shen, Ting Li, Nan Li, Yanyan Sun, Siyu Zhu, Nana Liu, Huifang Song, Kun Tang, Yujia Wang, Ying Zhang, Hui Cao, Yibo Wu, Yong Gan, Xinyao Zhang

**Affiliations:** 1 Department of Social Medicine and Health Management, School of Public Health, Jilin University, Changchun 130012, China; 2 Department of Social Medicine and Health Management, School of Public Health, Tongji Medical College, Huazhong University of Science and Technology, Wuhan 430030, China; 3 School of Humanities and Social Sciences, Harbin Medical University, Harbin 150081, China; 4 School of Public Health, Shaanxi University of Chinese Medicine, Xi’an 712046, China; 5 Department of Labor Economics and Management, Beijing Vocational College of Labour and Social Security, Beijing 100029, China; 6 School of Public Health, Peking University, Beijing 100083, China; 7 Health Culture Research Center of Shaanxi, Key Research Base of Philosophy and Social Sciences in Shaanxi Province, Xi’an 710016, China

**Keywords:** intention to pay for vaccination, intention, China, residents

## Abstract

Background: As an important part of the promotion of immunization programs and the suppression of infectious diseases, paid vaccines can prevent a variety of diseases and meet the needs of different populations. However, few studies focus on the public’s intention to pay for vaccination. Methods: The survey was conducted from 10 July to 15 September 2021, adopting a cross-sectional survey in China. We used a multi-stage sampling strategy to recruit participators from 120 cities. Participants filled out questions which assessed their intentions to pay for vaccination. A linear regression analysis was given to identify the predictors associated with the subjects’ attitudes. Results: There were 11,031 residents who finished our questionnaire. Chinese residents’ intention to receive paid vaccines scored 74.5 points. Residents who were male (*β* = −0.03) and older (30–44 (*β* = −0.03) or 45–59 (*β* = −0.06) or ≥60 (*β* = −0.08)), living alone (*β* = −0.03), who had moderate to severe anxiety (*β* = −0.03) or severe anxiety (*β* = −0.03) were more likely to refuse vaccination, while those who lived in Western China (*β* = 0.03) who had higher PSSS scores and HLS-SF12 index might acquire the intention to pay for vaccination. Conclusions: The study found that gender, age, region, living alone, anxiety, social support, and health literacy were the main influencing factors of residents’ attitudes. Governments and health institutions should take targeted measures to improve the health literacy and mental health of the population in order to facilitate the implementation of vaccination withdrawal and immunization policies.

## 1. Background

Vaccination is one of the most effective and cost-optimal interventions to improve the health of the population [1]. China began to implement planned immunization in 1978, expanded it to an immunization program in 1999, and then expanded the national immunization program in 2007 [2]. Through long-term immunization planning strategies, China has achieved the goal of significantly reducing the incidence of infectious diseases such as measles and hepatitis A. In 2016, the Chinese government updated the Regulations on the Administration of Vaccine Circulation and Vaccination [3] and stipulated that vaccines should be divided into free and paid vaccines. Free vaccines, also known as Category I vaccines, are vaccines that are included in the immunization program and are provided free of charge by the government to citizens. At present, the Chinese government has stipulated 15 kinds of vaccines such as hepatitis A and hepatitis B. Moreover, vaccines for COVID-19 were also provided to residents as free vaccines in the pandemic [4]. Paid vaccines outside the immunization program, also known as Category II vaccines, are administered by citizens at their own expense and on a voluntary basis. As a supplement to free vaccines, paid vaccines provide protection against a wide range of diseases and meet the needs of different populations [5,6,7].

Common paid vaccines include the influenza vaccine, chickenpox vaccine, rotavirus vaccine, rabies vaccine, and many others. The inoculation of paid vaccines is one of the effective measures for preventing infectious diseases. Studies have shown that the pneumonic conjugate vaccine, rotavirus vaccine, varicella vaccine, and other national Category II vaccines have obvious effects on the prevention of such infectious diseases [8,9,10]. However, these vaccines are expensive and have to be paid for by the population, which must be voluntary. Therefore, inhabitants’ intentions to get vaccinated are critical to determine whether paid vaccines can be widely used and whether infectious diseases can be contained.

Social science researchers have shown in past outbreaks that vaccine intention has complex causes. Individual psychological perception and access to health services play an important role. When thinking about how to encourage people to get vaccinated, the target audience’s community, the current environment, and the motivation that motivates people to get vaccinated are all important issues to consider. In China, the rate of free vaccination has reached a high level, while the rate of intention to pay for vaccination remains low [11]. Previous studies have examined the intentions of residents to receive free vaccines and COVID-19 vaccines [12,13,14], but few studies have focused on paid vaccines. Currently, many countries with poor economic foundations, especially developing countries, do not have access to free supplies of vaccines. As the most populous developing country, Chinese people’s intention to receive paid vaccines and its influencing factors can provide a reference for global immunization planners and researchers. This study investigates this topic and can help promote global access to vaccines and reduce the incidence of infectious diseases.

## 2. Methods

### 2.1. Ethics Statement

This study scheme was approved by the Institutional Review Committee of Ji’nan University, Guangzhou, China (JNUKY-2021-018). All methods were performed in accordance with relevant guidelines and regulations. Respondents were informed that their participation was voluntary.

### 2.2. Study Participants and Survey Design

The survey was conducted from 10 July 2021 to 15 September 2021. The study used multi-stage sampling. The capital cities of 33 provinces were included in the research. A total of 120 cities, 2–6 cities of each province by random number table method, were selected. Based on the “Seventh National Population Census in 2021”, investigators were openly recruited in these cities or survey groups, and 120 urban residents were selected for a fixed sample. According to population characteristics, samples were obtained by gender, age, and urban-rural distribution. At least one investigator or one investigation team was recruited in each city, each investigator was responsible for collecting 30–90 questionnaires, and each investigation team was responsible for collecting 100–200 questionnaires.

The surveyors used the network wenjuanxing platform, the most popular survey software in China (https://www.wjx.cn/, accessed on 10 July 2021), to issue questionnaires to the public in their respective areas. The respondents clicked the link to answer the questionnaire. The informed consent of the subjects was obtained during the survey, and the surveyors input the questionnaire number. If the respondent had thinking ability but not enough action ability to answer the questionnaire, the investigator would conduct a one-to-one interview and answer the questions on his or her behalf.

Inclusion criteria for this study included: (1) age ≥ 12 years; (2) the nationality of the People’s Republic of China; (3) China’s permanent resident population (annual travel time ≤ 1 month); (4) voluntary participation in the study and filling in the informed consent form; (5) participators could complete the network questionnaire survey by themselves or with the help of investigators; and (6) participators could understand the meaning of each item in the questionnaire. Exclusion criteria included: (1) inconvenient movement, confusion, and mental disorders; (2) those who were participating in other similar research projects; and (3) people who were unwilling to cooperate.

### 2.3. Instruments

Through literature reading and collection, the potential influencing factors on vaccination intention in previous studies were identified, and the questionnaire was designed accordingly. This questionnaire focused on the health status of residents and its influencing factors, and the relevant parts of this study included sociodemographic information (e.g., region, age, gender, education level, and marital status), social support status, health literacy, depression, anxiety, and intention to acquire paid vaccines. Residents’ intentions to receive paid vaccines were self-reported by residents. A scale of 0 to 100 was used, with higher scores indicating stronger intentions. Residents chose scores according to their own intentions to reflect their levels of willingness.

The Perceived Social Support Scale (PSSS) was used to measure social support status. The PSSS is a 12-item self-reported assessment of perceived emotional support from friends, family, and significant others [15]. Respondents indicated their degrees of agreement with the items on a 7-point Likert-type scale ranging from “very strongly disagree” to “very strongly agree”. Items were summed to obtain scores between 12 and 84, with higher scores indicating greater support. The Cronbach’s alpha of the PSSS was 0.91.

The short-form health literacy questionnaire (HLS-SF12) was used to measure health literacy (HL) [16]. People rated the perceived difficulty of each item on a four-point Likert scale (1 = very difficult, 2 = difficult, 3 = easy, and 4 = very easy). The indices for HL were standardized to unified metrics from 0 to 50 using the formula: Index = (mean − 1) × (50/3), where Index is the specific index calculated, mean is the mean of all participating items for each individual, 1 is the minimal possible value of the mean (leading to a minimum value of the index of 0), 3 is the range of the mean, and 50 is the chosen maximum value of the new metric. Thus, an index value was obtained, where 0 represents the lowest HL and 50 the highest HL [17]. The higher index reflects the stronger health literacy of respondents. The Cronbach’s alpha of the HLS-SF12 was 0.87.

The Patient Health Questionnaire (PHQ-9) was used to assess depressive disorders, using nine items scored on their frequency, from “not at all” to “nearly every day”, resulting in a score between 0 and 27 (higher scores representing greater depressive symptoms) [18]. This items aligned with the DSM-V criteria for identifying depressive disorders. The PHQ-9 scores were also used to categorize the severity of depression symptoms: scores of 0–4 were no symptoms, 5–9 were mild, 10–14 were moderate, 15–19 were moderately severe, and 19–27 were severe symptoms. The Cronbach’s alpha of the PHQ-9 was 0.84.

The 7-item generalized anxiety disorder (GAD-7) was used to measure anxiety status, using a 7-item self-report instrument which widely assessed generalized anxiety disorder based on DSM-IV criteria [19]. Anxiety is a feeling of fear, dread, and uneasiness. It might cause sweating, feeling restless and tense, and having a rapid heartbeat. It can be a normal reaction to stress. Anxiety is a generalized symptom that can be emotionally directed against anyone or anything which is not about the COVID-19 vaccine. Each item was scored on a 4-point Likert scale indicating symptom frequency, ranging from 0 (not at all) to 3 (nearly every day). The GAD-7 total score could range from 0 to 21. Scores of 0–4 were no symptoms, 5–9 were mild, 10–13 were moderate, 14–18 were moderately severe, and 19–21 were severe symptoms. The Cronbach’s alpha of the GAD-7 was 0.87.

### 2.4. Statistical Methods

The data were analyzed using *SPSS™* for Windows, Version 22.0 (SPSS, Inc., Chicago, IL, USA). Data analysis included the mean and standard deviation of continuous variables and the quantity and percentage of classified data. The *t*-test and ANOVA were used to compare differential factors for vaccination intentions scores. In addition, the intentions scores of residents in different regions were analyzed by stratification. No clustering was observed in the respondents (correlation = 0.03, *p* < 0.001). Therefore, the multivariable linear stepwise regression analysis model was used to estimate the factors associated with vaccination intentions scores in residents. *p* values were evaluated using sequential Bonferroni correction analyses. All comparisons were two-tailed and a *p* value of <0.05 was defined as statistically significant.

## 3. Results

Table 1 presents the main characteristics of the survey respondents. Among the 11,031 respondents, most were women (54.37%) and had a college degree or above (58.81%). The mean age (SD) was 31.66 (8.06) years. Among the respondents, 5609 (50.85%), 2993 (27.13%), and 2429 (22.02%) were from Eastern, Central, and Western China, respectively. More than half of the participants were unemployed (57.96%) and lived in urban settings (72.60%) (Table 1). The average score of the residents’ perceived social support scale was 60.220 ± 13.026, and the average index of the health literacy scale was 34.308 ± 8.393. In addition, residents’ intention to receive paid vaccines was 74.42 ± 27.30 points.

The factors associated with the intention to pay for vaccination intentions are presented in Table 2. Residents who were male (*β* = −0.03) and older (30–44 (*β* = −0.03) or 45–59 (*β* = −0.06) or ≥60 (*β* = −0.08)), living alone (*β* = −0.03), who had moderate to severe anxiety (*β* = −0.03) or severe anxiety (*β* = −0.03) were more likely to refuse vaccination, while those who lived in Western China (*β* = 0.03) who had higher PSSS scores and HLS-SF12 indexes might acquire the intention to pay for vaccination (Table 2). In addition, we stratified the study sample by region and conducted regression analyses. The results show that residents who have college degrees or above educational levels (*β* = 0.06) and high income (per capita monthly household > 12,000, *β* = 0.11) are more likely to receive paid vaccines in Western China (Table 3).

## 4. Discussion

This study examined the intention of Chinese residents to pay for vaccination and its influencing factors. The result showed that the average score of residents was 74.42, which was at a high level, indicating that residents had a high intention to have these vaccines. In addition, the study also identified some factors that might influence intention, which could provide a reference for targeted vaccine promotion strategies.

Firstly, gender is a factor that affects the intention of residents. Women were more likely than men to receive the intention to pay for vaccination, consistent with previous research. The study of Deng et al. showed that female college students were more likely to be vaccinated against human papillomavirus (HPV) than their male counterparts [20]. Luk et al.’s research also showed that men were less receptive to the COVID-19 vaccine [21]. Moreover, there was also a higher intention among older residents to receive the intention to pay for vaccination. Lu et al.’s research showed that older people were more likely to be vaccinated against COVID-19 than younger people [22]. This finding of Qiang et al. also found a similar result [23], reflecting higher predicted health risks in older adults [22].

A previous study found that vaccination intention was not associated with health literacy [24]. However, our research shows that there is a significant correlation between the health literacy of the population and the intention to pay for vaccination. Residents who are averse to their own health risks are more likely to receive more vaccinations to prevent potential health risks [25]. This finding is consistent with the hypothesis of health behavior theory [26]. Another interesting finding is that vaccination was significantly associated with social support, with residents with higher PSSS scores more likely to develop intention, while solitary residents might be less willing. This suggests that adequate social support can increase the willingness of the population to safeguard their health and prevent disease through vaccination. In addition, residents with higher anxiety levels were more likely to be vaccinated, which was also linked to their expected health risks.

Notably, there was no significant correlation between income and the intention to get vaccinated. The results of this study also indicate that the residents of the western region still have a higher willingness to get vaccinated, although the economic foundation of the western region is relatively weak. Therefore, economic factors may not be the critical factor in the population’s decision to get vaccinated, but the spread of vaccines may be related to the intensity of local promotion, as well as the population’s age structure and health awareness [27]. In the analysis of residents in Western China, high education levels can promote the enhancement of vaccination intention, which may be related to the cognitive differences of residents with different educational backgrounds [28].

“Unemployment” rates were higher among participants in this study but not directly related to intention. In fact, they were related to the way we conducted our investigations. This study used a web-based method to collect questionnaires. In China, young people are the main community of web-users, especially students. Therefore, the majority of those who participated in our questionnaire were students, and they were unemployed. Many student groups are college students, so they are out of employment. Because of this offset, employment may not be clearly linked to intention. The study presents a high rate of “unemployment”, but this element itself may not have a clear link with intention.

In conclusion, this study analyzed the intention to receive paid vaccines and related factors, especially finding that psychological factors and health pursuits were the key variables for improving the willingness to get vaccinated. However, inadequate health education remains an important barrier to vaccination in developing countries [29]. Therefore, countries should focus on strengthening the education of vaccination knowledge publicity, increasing the access of residents to health information, and promoting the training and education of the skills of medical personnel on vaccination.

### Strengths and Limitations

This study discussed the public intention to pay for vaccination. We used a nationwide sample of the Chinese population to explore the related factors. The perspective of this study can provide some reference for future research on vaccination policy theory and help researchers better understand the process of paid vaccine promotion.

However, this study has some limitations. First, as the behaviors were self-reported, reporting bias was possible. Second, the distribution of the study participants was imbalanced across regions (5609:2993:2429); therefore, the subgroups of variables might not be representative of the population. 

The self-selection and social desirability biases had potential effects on the outcome. This study could not determine how many participants reviewed the online poster or survey but decided not to complete the survey; thus, the presence of non-response bias could not be assessed. Overall, the generalization of the results should be regarded with caution.

The present study lacks a systematic study of the deeper causes. Different social groups have different definitions of the concepts of immunization and vaccine efficacy, and these definitions influence their attitudes toward vaccination. Such differences exist between countries and even within countries. For example, some social groups may view vaccines as being too “powerful”, believing that they attack the body and hinder natural immunity. There are also social groups that are concerned about vaccine antigens and adjuvants, arguing that they are toxic or too immunogenic. In conclusion, both self-selection and social desirability bias could have an impact on willingness to get vaccinated and need to be further investigated.

Even though COVID-19 vaccines are offered as free vaccines and this study focuses on those that are paid for by the public, the findings of this study still have some value. The findings of this study on the potential factors affecting residents’ intentions to get vaccinated also have virtual implications for the public’s acceptance of the new coronary pneumonia vaccine. Therefore, this study has implications for the general vaccination and promotion of COVID-19 vaccines in the future. 

In times of uncertainty, people may make decisions about whether to get vaccinated based on their own judgments about disease risk rather than vaccination risk. Hesitancy for COVID-19 vaccination can undermine public health benefits. A variety of complex social, political, economic, and cultural factors that influence vaccine acceptance also conspire to influence COVID-19 vaccines. There are still many unknowns about COVID-19, which could undermine confidence in vaccines and make people wonder if a safe and effective vaccine might be successful. Although our study is not for a COVID-19 vaccine, it could still inform this question. The dynamics of disease outbreaks in different settings are different, affecting people’s risk assessment conclusions. The strategies and approaches proposed by this study can help policymakers, public health officials, vaccine developers, medical workers, and others build and maintain public confidence in a COVID-19 vaccine, thereby increasing vaccination rates. A multi-pronged approach, tailored to the sociopolitical environment and specific social groups or even individuals, may be the most effective way to increase confidence in a COVID-19 vaccine.

Future research could explore more factors that may influence residents’ opinions based on the present study, such as occupation or social culture. In addition, longitudinal studies should be conducted in the future to evaluate the relationship between various influencing factors and attitudes of intention to pay for vaccination among residents.

## 5. Conclusions

This study found that Chinese residents’ intention to receive paid vaccines scored 74.5 points and that gender, age, region, living alone, anxiety, social support and health literacy were the main influencing factors of residents’ attitudes. The promotion of the intention to pay for vaccination is closely linked to immunization programs. Therefore, at the national level, although the state cannot pay for Type II vaccines (because it is too expensive and will burden the national finances), effective measures such as publicity and education can still be used to make citizens pay for this vaccine voluntarily. Enhanced communication, dialogue, and collaborative planning with the community to explore the rollout and availability of vaccines can further help strengthen residents’ intentions to receive these vaccines.

## Figures and Tables

**Table 1:** Statistical description and vaccination intentions scores of study samples.

Variables	N (%)	M ± SD	*F*	*p* Value
Total	11,031 (100)	74.42 ± 27.30	NA	NA
Gender				
Male	5033 (45.63)	73.04 ± 27.36	23.71	<0.01
Female	5998 (54.37)	75.58 ± 27.19		
Age group, y				
<30	4665 (42.29)	76.93 ± 26.39	38.76	<0.01
30–44	3001 (37.21)	74.58 ± 26.51		
45–59	2218 (20.11)	72.16 ± 28.00		
≥60	1147 (10.40)	68.16 ± 30.09		
Region				
Eastern China	5609 (50.85)	73.91 ± 27.53	2.58	0.076
Central China	2993 (27.13)	74.61 ± 27.45		
Western China	2429 (22.02)	75.38 ± 26.53		
Highest educational level				
Primary school or below	1127 (10.22)	67.73 ± 31.19	43.83	<0.01
Middle school	3417 (30.98)	73.89 ± 27.18		
College degree or above	6487 (58.81)	75.86 ± 26.44		
Marital status				
Unmarried	4805 (43.56)	75.90 ± 26.99	25.10	<0.01
Married	6226 (56.44)	73.28 ± 27.48		
Place of residence				
Urban	8008 (72.60)	74.90 ± 27.20	8.91	0.003
Rural	3023 (27.40)	73.16 ± 27.52		
Whether living alone				
Yes	1271 (11.52)	71.04 ± 28.61	22.16	<0.01
No	9760 (88.48)	74.86 ± 27.09		
Whether having own career				
Yes	4637 (42.04)	75.36 ± 26.78	9.38	0.002
No	6394 (57.96)	73.75 ± 27.65		
Per capita monthly household income				
≤6000	7500 (67.99)	74.20 ± 27.50	1.24	0.290
6001–12,000	2769 (25.10)	75.13 ± 26.68		
>12,000	762 (6.91)	74.04 ± 27.45		
Whether having health insurance				
Yes	8732 (79.16)	74.70 ± 27.21	4.47	0.035
No	2299 (20.84)	73.35 ± 27.58		
Whether having chronic disease				
Yes	2047 (10.56)	70.90 ± 28.78	42.04	<0.01
No	8984 (81.44)	75.23 ± 26.88		
Number of medications taken daily				
0	8996 (81.55)	75.17 ± 26.87	22.66	<0.01
1–2	1540 (13.96)	72.16 ± 28.35		
≥3	495 (4.49)	67.95 ± 30.25		
Depression				
No depression	2982 (27.03)	76.28 ± 27.67	5.09	<0.01
Mild depression	1932 (17.51)	74.22 ± 26.76		
Moderate depression	1493 (13.53)	74.01 ± 27.24		
Moderate to severe depression	2290 (20.76)	74.18 ± 26.79		
Severe depression	2334 (21.16)	72.72 ± 27.66		
Anxiety				
No anxiety	2982 (27.03)	75.90 ± 27.47	18.81	<0.01
Mild anxiety	1932 (17.51)	73.76 ± 26.99		
Moderate anxiety	1493 (13.53)	68.28 ± 26.22		
Moderate to severe anxiety	2290 (20.76)	70.16 ± 26.90		
Severe anxiety	2334 (21.16)	76.40 ± 27.36		

**Table 2:** Stepwise regression analysis of associated factors for vaccination intentions scores among respondents.

Variables	Unstandardized Coefficients		Standardized Coefficients	*t*	*p*	95% CI
	*β*	*SE*	*β*			
Gender (ref: female)						
Male	−1.52	0.51	−0.03	−2.97	0.01	−2.52–−0.52
Age group, y (ref: <30)						
30–44	−2.04	0.62	−0.03	−3.27	0.01	−3.26–−0.82
45–59	−4.37	0.69	−0.06	−6.31	<0.01	−5.73–−3.01
≥60	−6.75	0.90	−0.08	−7.53	<0.01	−8.51–−5.00
Region (ref: Eastern China)						
Western China	1.70	0.61	0.03	2.78	0.01	0.50–2.89
Whether living alone (ref: no)						
Yes	−2.40	0.80	−0.03	−2.99	0.01	−3.97–−0.83
Anxiety (ref: no anxiety)						
Moderate to severe anxiety	−2.93	0.99	−0.03	−2.95	0.01	−4.88–−0.98
Severe anxiety	−3.83	1.18	−0.03	−3.25	0.01	−6.14–−1.52
PSSS score	0.24	0.02	0.11	10.91	<0.01	0.19–0.28
HLS-SF12 index	0.40	0.03	0.12	11.71	<0.01	0.33–0.47

**Table 3:** Stratified stepwise regression of predictors of vaccination intentions scores by region.

Variables	Unstandardized Coefficients		Standardized Coefficients	*t*	*p*	95% CI
	*β*	*SE*	*β*			
Eastern China						
Gender (ref: female)						
Male	−1.91	0.73	−0.03	−2.62	0.01	−3.33–−0.48
Age group, y (ref: <30)						
45–59	−3.78	0.90	−0.06	−4.20	<0.01	−5.54–−2.01
≥60	−4.90	1.23	−0.05	−3.98	<0.01	−7.31–−2.48
Whether living alone (ref: no)						
Yes	−3.42	1.13	−0.04	−3.02	0.01	−5.64–−1.20
PSSS score	0.22	0.03	0.11	7.32	<0.01	0.16–0.28
HLS-SF12 score	0.42	0.05	0.13	8.65	<0.01	0.32–0.51
Central China						
Gender (ref: female)						
Male	−2.42	0.99	−0.04	−2.45	0.01	−4.36–−0.48
Whether living alone (ref: no)						
Yes	−4.34	1.57	−0.05	−2.76	0.01	−7.42–−1.26
PSSS score	0.25	0.04	0.12	5.80	<0.01	0.16–0.33
HLS-SF12 score	0.32	0.06	0.10	4.93	<0.01	0.19–0.44
Western China						
Age group, y (ref: <30)						
≥60	0.09	0.03	0.07	3.20	0.01	0.03–0.14
Highest educational level (ref: primary school or below)						
College degree or above	0.06	0.02	0.06	2.90	0.01	0.02–0.10
Whether living alone (ref: no)						
Yes	0.14	0.03	0.09	4.08	<0.01	0.07–0.20
Per capita monthly household income (ref: ≤6000)						
>12,000	0.26	0.05	0.11	5.57	<0.01	0.17–0.35
PSSS score	−0.01	0.01	−0.06	−2.92	0.01	−0.01–−0.01
Anxiety (ref: no anxiety)						
Severe anxiety	−0.07	0.02	−0.06	−3.00	0.01	−0.12–−0.02

## Data Availability

Data may be made available by contacting the corresponding author.
